# Development of an Innovative Soft Piezoresistive Biomaterial Based on the Interconnection of Elastomeric PDMS Networks and Electrically-Conductive PEDOT:PSS Sponges

**DOI:** 10.3390/jfb13030135

**Published:** 2022-08-29

**Authors:** Maria Antonia Cassa, Martina Maselli, Alice Zoso, Valeria Chiono, Letizia Fracchia, Chiara Ceresa, Gianluca Ciardelli, Matteo Cianchetti, Irene Carmagnola

**Affiliations:** 1 Department of Mechanical and Aerospace Engineering, Politecnico di Torino, Corso Duca degli Abruzzi 24, 10129 Torino, Italy; 2 Polito BIOMed Lab, Politecnico di Torino, Corso Castelfidardo 30/a, 10129 Torino, Italy; 3 The BioRobotics Institute, Scuola Superiore Sant’Anna, Viale Rinaldo Piaggio 34, 56025 Pontedera, Italy; 4 Department of Excellence in Robotics & AI, Scuola Superiore Sant’Anna, Piazza Martiri della Libertà 33, 56127 Pisa, Italy; 5 Institute for Chemical and Physical Processes (CNR-IPCF), National Research Council, 56124 Pisa, Italy; 6 Department of Pharmaceutical Sciences, Università del Piemonte Orientale “A. Avogadro”, Largo Donegani 2, 28100 Novara, Italy

**Keywords:** biomaterials engineering, piezoresistive material, soft and flexible transducer, interconnected networks

## Abstract

A deeply interconnected flexible transducer of polydimethylsiloxane (PDMS) and poly(3,4-ethylenedioxythiophene):polystyrenesulfonate (PEDOT:PSS) was obtained as a material for the application of soft robotics. Firstly, transducers were developed by crosslinking PEDOT:PSS with 3-glycidyloxypropryl-trimethoxysilane (GPTMS) (1, 2 and 3% *v*/*v*) and using freeze-drying to obtain porous sponges. The PEDOT:PSS sponges were morphologically characterized, showing porosities mainly between 200 and 600 µm^2^; such surface area dimensions tend to decrease with increasing degrees of crosslinking. A stability test confirmed a good endurance for up to 28 days for the higher concentrations of the crosslinker tested. Consecutively, the sponges were electromechanically characterized, showing a repeatable and linear resistance variation by the pressure triggers within the limits of their working range ($\frac{∆R}{R_{0}}\;$ max = 80% for 1–2% *v*/*v* of GPTMS). The sponges containing 1% *v*/*v* of GPTMS were intertwined with a silicon elastomer to increase their elasticity and water stability. The flexible transducer obtained with this method exhibited moderately lower sensibility and repeatability than the PEDOT:PSS sponges, but the piezoresistive response remained stable under mechanical compression. Furthermore, the transducer displayed a linear behavior when stressed within the limits of its working range. Therefore, it is still valid for pressure sensing and contact detection applications. Lastly, the flexible transducer was submitted to preliminary biological tests that indicate a potential for safe, in vivo sensing applications.

## 1. Introduction

Soft robotics is one of the most innovative branches of robotic engineering, aiming to design and produce mechanical robots and actuation systems that take inspiration from human and animal interactions with the natural environment, overcoming the typical rigidity that characterizes robots [1]. Consequently, all of the materials employed are soft, stretchy, and deformable; for example, viscoelastic polymeric elastomers, mainly polysiloxanes, are used given their safe interaction with biological organisms [2]. The biomedical applications of soft robotics involve soft tools for surgery, diagnosis, drug delivery, but also other applications, such as prostheses, wearable assistive devices, artificial organs, and tissue-mimicking systems that could either be used for medical training or in-depth studies as body-part simulators [3]. All of the above-mentioned cases make extensive use of sensing elements, whether it is through force/contact detection or strain, torsion, or displacement transduction. Hence, there is a demand for soft and flexible transducers, based on elastomer-conductive composite materials and able to combine mechanical elasticity and deformability with electrical conductivity and responsivity. Flexible sensors can be sorted based on their working mechanism, mainly as capacitive, piezoelectric, and piezoresistive [4]. Piezoresistive soft sensors are of particular interest because of their high sensitivity, low cost, ease of production, fast response, and easy signal acquisition. Over the last few years, hydrogel-based [5,6,7], polyurethane-based [8,9], and PDMS-based [10,11,12] piezoresistive flexible sensors, which combine an elastic matrix with conductive fillers, have been widely investigated. Typically, nanosized inorganic conductive fillers have been proven to successfully transfer their electrical properties to elastomeric matrices: carbon nanotubes [13,14], metallic nanoparticles [15], carbon black [16], carbon fibres [17], and several graphite derivatives [13,18] have been widely investigated and employed. Their efficacy is attributed to their nanometric dimensions, aside from their electronic structure, which allow for a greater exposed specific surface area and therefore a greater exchange at the interface between matrix and filler; this is essential to the effective transfer of electrical properties to the elastomeric matrix [19]. Metallic and carbon-based materials, however, hardly match the viscoelastic properties and mechanical behaviour of silicone rubbers and elastomers, often increasing their elastic modulus and hardness [13], thus making these composites less suitable for soft robotics applications. A possible improvement is the employment of an organic conductive filler to better match the viscoelastic properties of the matrix. Following this strategy, an interesting class of materials is constituted by conductive polymers, such as polyaniline (PANI) [20,21,22], polypyrrole (PPy) [23,24], and poly(3,4-ethylenedioxythiophene) (PEDOT) [25,26]. In particular, the combination of PEDOT with polystyrenesulfonate (PSS) is impressive, as its intrinsic electrical conductivity (0.2–1 S/cm), which strongly depends on the PEDOT:PSS ratio, can be further optimized using a wide range of secondary dopants; this allows for increases in conductivity magnitude as much as 4 orders greater [27,28]. Moreover, the conjugation of PEDOT and PSS yields a conductive polymer that is easily solubilized in water and therefore more suitable for industrial processing through low-cost and well-established production techniques [29]. Usually, the combination of silicone elastomers such as PDMS with conductive polymers involves some form of layering, where the conductive polymer is imprinted, electrospun, spin-coated, or polymerized on the silicone surface, forming a conductive pattern. [25,30,31,32,33] These methodologies end up creating a conductive coating layer, but the flexible substrate beneath remains intrinsically non-conductive. Due to the extremely different natures, it is very difficult to obtain homogeneous PDMS and PEDOT:PSS blends; however, some efforts have been made to improve their miscibility. Luo et al. [28] used an amphiphilic surfactant P-t-octylophenol (Triton X-100) to reduce the hydrophobicity of PDMS and successfully blend it with PEDOT:PSS, yielding stretchable conductive polymer films with stable electrical properties. In the work by Noh [34], a block co-polymer of hydrophobic PDMS backbones and hydrophilic PEO side chains (PDMS-b-PEO) was introduced into the PDMS and PEDOT:PSS mixture in order to minimize the interfacial tension between the two different phases in the blend and increase their dispersion. Such a strategy found a promising application in printable electronics [35] for use on stretchable knitted cotton fabric via flat-screen printing for the production of textile-based sensors and/or electrodes. In this study, the goal was to fabricate a soft piezoresistive transducer by producing conductive polymer-based sponges, which vary their intrinsic resistance value in response to the mechanical compression triggers, therefore allowing their usage in force/contact detection applications. To this aim, a deep interconnection between PDMS and PEDOT:PSS was obtained using only processing techniques without the use of chemical modifications and/or additives, yielding an altogether original material that improved the PEDOT:PSS sponge fragility and stretchability while preserving their piezoresponsiveness. The conductive polymer, processed in an aqueous solution, was primarily crosslinked using three different concentrations of 3-glycidyloxypropryl-trimethoxysilane (GPTMS) and then freeze-dried in order to produce PEDOT:PSS sponges. Thereafter, the sponges were completely immersed in a solution of PDMS oligomer and curing agent so that the elastomer could infiltrate the porosities and yield a network by in situ polymerization. The native PEDOT:PSS sponges have been morphologically and electromechanically characterized. The composite sponge results demonstrated that the flexible, stretchable, water stable, and piezoresponsive characteristics were preserved. The combination with PDMS does not significantly lower PEDOT:PSS native sensitivity, making this strategy a promising and feasible possibility for the realization of stretchable organic interconnects that could be used as contact detection sensors in a wide range of biomedical applications, as soon as a calibration curve of the developed sensor is acquired.

## 2. Materials and Methods

### 2.1. Materials

The aqueous solution of poly(3,4-ethylenedioxythiophene) polystyrenesulfonate (PEDOT:PSS) was purchased from Ossila. 3-glycidyloxypropryl-trimethoxysilane (GPTMS) with a molecular weight of 236.34 g/mol. was purchased from Sigma-Aldrich (Milano, Italy). PDMS (Polydimethylsiloxane) discs were obtained from the SYLGARD 184 Silicone Elastomer Kit supplied by the Dow Chemical Company (Midland, MI, USA).

### 2.2. Preparation of Piezoresistive Sponges

The PEDOT:PSS sponges were obtained using the freeze-drying technique. A PH1000 solution was combined with crosslinker GPTMS at three different concentrations, at 1, 2, and 3% *v*/*v*. The samples will henceforth be referred to as PEDOT_GPTMS 1%, PEDOT_GPTMS 2%, and PEDOT_GPTMS 3%. 3 mL of these solutions were poured in circular moulds (Ø = 3.5 mm, thickness = 3 mm) and immediately frozen at −20 °C for at least 5 h. The samples were then completely freeze-dried using a CoolSafe freeze-dryer (SCANVAC) for 24 h, obtaining macroporous, sponge-like constructs.

### 2.3. Preparation of Flexible Transducer

To fabricate the flexible transducers, the conductive PEDOT_GPTMS 1% sponges were combined with an elastomeric silicone, using a volume ratio of 2:1 (PEDOT:PSS to silicone, respectively). A silicone precursor solution was obtained by mixing the two liquid components (the base and curing agent) with a weight ratio of 10:1 for PDMS. The solution was briefly degassed in order to eliminate the air entrapped during the mixing phase; the total volume was then equally divided into two separate wells. The PEDOT:PSS freeze-dried sponges were initially immersed in the first well for 5 min (allowing the silicone precursor to infiltrate the pores), then the specimens were flipped and immersed in the second well for another 5 min. Finally, the samples were retrieved and left to polymerize for the remainder of the polymerization interval (48 h for PDMS) at room temperature. Such samples will be henceforth referred to as PEDOT_GPTMS 1%/PDMS.

### 2.4. Morphological Analysis

Images of the specimen surfaces and cross-sections were obtained using a scanning electron microscope (Zeiss LEO 435VP, SEM, Jena, Germany) at different magnifications. Before analysis, samples were given a thin gold coating. Images were then elaborated through the ImageJ software by NIH in order to estimate the pore diameters.

### 2.5. Water Contact Angle Measurement (WCA)

The sample wettability was estimated through contact angle measurement using the Drop Shape Analyzer DSA25 (Krüss) device, paired with the Advance software (Krüss GmbH, Hamburg, Germany). The static WCA was obtained via the sessile droplet method, depositing 2 μL drops of Milli-Q water on the sample. The results of the wettability test were compared with the native PDMS behaviour.

### 2.6. Mechanical Tests

The flexible transducers were also subjected to pure compression mechanical tests, carried out in triplicates with the Instron material testing machine (Model 4464, Instron Inc., Norwood, MA, USA); the tests included a 1 kN strain gauge load cell and a cubic compressor surface area of 4 cm^2^. The results of the mechanical characterizations were compared with both the native PEDOT:PSS and PDMS behaviour.

Four compression cycles were applied at a constant crosshead speed of 0.5 mm/min to a maximum load (corresponding to 2.4 mm of the maximum compression displacement). Three replicates were performed for each PEDOT:PSS sponge and flexible elastomer/PEDOT:PSS specimen. The mean values and the standard deviations (SD) of the mechanical hysteresis and maximum stress were considered for each replicate. The results were then averaged on the three trials and the SDs were considered.

### 2.7. Stability Evaluation

The native PEDOT:PSS samples underwent a stability test in an aqueous environment. They were weighted (w_o_), subsequently immersed in phosphate-buffered saline (PBS, pH 7.4) at 37 °C to mimic the physiological conditions, and evaluated at 6 different time intervals (1, 3, 7, 14, 21, and 28 days), replacing the used PBS solution with a fresh solution every 3 days. At the end of each time interval, the samples were thoroughly washed with distilled water 3 times and then frozen overnight. Ultimately, the samples were freeze-dried and again weighted (w_i_), where their percentages of lost/retained weight were calculated as follows:

(1) $$\%\;\mathrm{weight}\;\mathrm{loss}\;(\mathrm{WL})=\frac{W_{o}-W_{i}}{W_{o}}\cdot 100$$

(2) % retained weight (RW) = 100 − WL

### 2.8. Determination of Conductivity and Piezoresistive Behaviour

The preliminary measurements of impedance were performed using the Agilent E4980A Precision LCR meter on both the native PEDOT:PSS sponges and flexible elastomer/PEDOT:PSS specimens. The piezoresistive behaviour of all the above-mentioned samples was further investigated by determining the relationship between the mechanical stress (σ) applied to the material and the variation of its resistance value ($\frac{∆R}{R_{0}}$), where *R*_0_ is the resistance at zero value stress. The electromechanical tests performed involve the use of the Instron material testing machine paired with a simple voltage divider circuit and a data acquisition board (DAQ National Instruments^®^, see Supplementary Information Figure S1 for the instrumentation setup); the samples were connected to the wires by soldering wires on copper tape. Both the mechanical and electrical data were acquired via the LabView (Laboratory Virtual Instrumentation Engineering Workbench, Austin, TX, USA) software developed by National Instruments, and their acquisition was synchronized by setting the same sampling frequency (fc = 10 Hz). The sample was integrated within a voltage divider circuit as the unknown resistor in series, with a fixed resistor of an appropriate resistance value (R_c_); the value of the R_c_ was estimated for each sample according to the preliminary impedance measured though the LCR meter previously mentioned. The value of the R_c_ should always be as close as possible to the unknown resistor to guarantee the optimal functioning of the voltage divider circuit. The voltage divider can be exploited as a method to determine the value of an unknown resistor (R_sample_) in the circuit given the circuit transfer function (V_in_ = input tension, V_out_ = output tension) (Equation (3))

(3) $$H=\frac{V_{\mathrm{out}}}{V_{\mathrm{in}}}=\frac{R_{\mathrm{sample}}}{R_{c}+R_{\mathrm{sample}}}$$

where R_sample_ can be calculated knowing R_c_, V_in_, and measuring V_out_ using Equation (4):

(4) $$R_{\mathrm{sample}}=R_{c}\frac{V_{\mathrm{out}}}{V_{\mathrm{in}}-V_{\mathrm{out}}}\;\left[\Omega \right]$$

The parameters of each voltage divider circuit employed during the study are summarized in Table 1.

Before starting any mechanical test, the initial resistance value R_sample_ was registered. It was then fixed to the Instron testing platform, where we set the parameters of the mechanical test, such as the number of cycles, velocity of deformation, and maximum strain applied (Table 2). All of the tests carried out during the study were compressive, cyclic, and performed in triplicates.

Six load values, equally spaced from 0 to 30 kPa, were applied on a square area of the transducer using a rectangular indenter (20 × 20 mm^2^) at a constant crosshead speed of 10 mm/min. Four cycles were performed for each test. The mean values and the standard deviations (SD) of the resistance variation were considered for each replicate; then, the results of the three trials were averaged and the SDs were considered to evaluate the repeatability of the piezoresistive behaviour.

### 2.9. Evaluation of Cytotoxicity

A test of direct cytotoxicity was carried out following the ISO 10993-5:2018 document, using NIH 3T3 cells (ATCC*^®^*CRL-1658^TM^). The NIH 3T3 cells were cultured in a 96-plate multiwell, using a cell density of 2 *×* 10^4^ cells/well. The medium for cellular cultures was produced in the following composition: 90% of commercial DMEM (Dulbecco modified Eagle medium), 10% of FBS (fetal bovine serum), 1% of glutamine, and 1% of antibiotics such as penicillin and streptavidin. After 24 h, the cells reached confluence; the culture medium was removed from each well and substituted with the conditioned medium, which was prepared by soaking the flexible transducer samples in DMEM at a ratio of 1 mL:0.1 g for 24 h at 37 °C. Control samples (CTRL) were prepared by substituting the medium with unconditioned fresh medium. After 24 h, the medium was substituted in each well with 100 µL of 0.1 mg/mL non-fluorescent resazurin solution in phosphate-buffered saline (PBS). The cell viability was measured through the fluorimetry analysis as non-fluorescent resazurin was reduced to highly fluorescent resorufin via cell metabolism once it entered the mitochondria, and the fluorescent signal was monitored using a plate reader device (Biotek, Winooski, VT, USA) at a 530 nm excitation wavelength and 590 nm emission wavelength. Four different samples were weighted and tested from both the PDMS and flexible transducer. Higher fluorescence determines higher concentrations of resofurin and, therefore, higher metabolic activity of the cells: this signifies a higher viability, and consequently poorer cytotoxicity of the material. The emission was detected using another plate reader device (Victor3V^TM^, Perkin Elmer, Milano, Italy) and the intensity of the signal is directly proportional to the number of viable cells. The results have been reported in terms of mean ± standard deviation; the mean was calculated first among the three repetitions and second among the four samples tested.

### 2.10. Activity against S. aureus and C. albicans Biofilms

A *Staphylococcus aureus* ATCC 6538 suspension was prepared at a cell density of 1 × 10^7^ CFU (colony-forming unit)/mL in tryptic soy broth (Scharlab, Barcelona, Spain) that was supplemented with 1% glucose (Biolife, Milano, Italy). A *Candida albicans* ATCC 10,231 suspension was prepared at a cell density of 1 × 10^6^ CFU/mL in RPMI 1640 medium (Sigma-Aldrich) that was supplemented with 1% glucose. The PDMS and PEDOT_GPTMS 1%/PDMS discs were submerged in 1 mL of microbial suspensions and incubated at 37 °C in static conditions. After 24 h, the growth medium was removed, and the discs were gently washed with 1 mL of PBS to remove non-adherent cells. The anti-biofilm effect of the PEDOT_GPTMS 1%/PDMS discs was evaluated by crystal violet (CV) staining: biofilms were dried and stained with 1 mL of CV solution (0.1%) for 10 min. Blank discs (without biofilm) were also included. After the removal of excess dye, biofilms were air-dried and then CV solubilized with 1 mL of acetic acid (33% in water). The solution absorbance at 570 nm was registered to estimate the amount of CV and, therefore, the biofilms’ biomass. Supernatants corresponding to the PDMS and PEDOT_GPTMS 1%/PDMS discs were collected after centrifugation (17000 rpm for 15 min), and the discs were incubated in 1 mL of MTT working solution (0.075% MTT solution supplemented with 0.1% glucose and 10 µM of menadione) for 30 min. Afterwards, violet formazan crystals were dissolved with 1 mL of lysis solution (DMSO/0.1 M glycine buffer (pH 10.2) solution (7:1)) [36]. Once again, the absorbance of the solution was measured at 570 nm (A570) (Victor3V^TM^, Perkin Elmer, Italy) and data were normalized to the blanks. The percentages of inhibition were calculated with the following formula:

(5) $$\%\;\mathrm{inhibition}=(1-A_{\mathrm{treat}}/A_{\mathrm{ctrl}})\;\times \;100\;$$

where $A_{\mathrm{treat}}$ is the absorbance of the PEDOT_GPTMS 1%/PDMS discs and $A_{\mathrm{ctrl}}$ is the absorbance of the PDMS discs.

## 3. Results and Discussion

**Figure 1:**
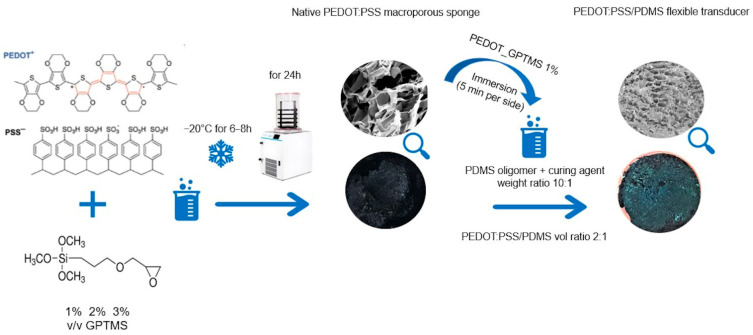
Flow chart of the fabrication steps for both the native PEDOT:PSS sponges and PEDOT:PSS/PDMS flexible transducers.

In this study, piezoresistive sponges were produced through the freeze-drying of crosslinked PEDOT:PSS aqueous solution and were broadly characterized in terms of their porosities distribution, stability, and electromechanical properties. These sponges were later combined with an elastomeric network of PDMS in order to improve their brittleness and stretchability; the novel interconnected transducer surface wettability, mechanical behaviour, piezoresponsivity, cytotoxicity, and anti-biofilm properties were investigated. A schematic representation of the processes involved is given in Figure 1.

### 3.1. Distribution of Porosities

**Figure 2:**
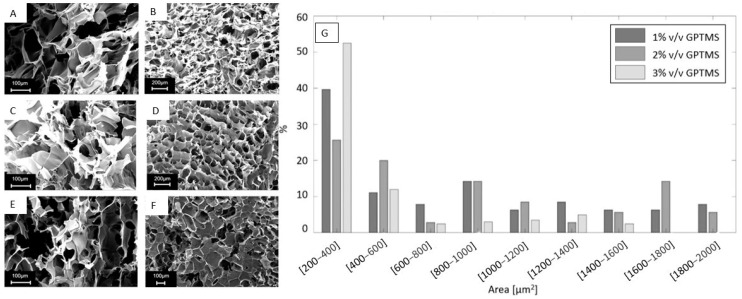
(**A**) PEDOT_GPTMS 1% SEM image under 250 times magnification. (**B**) PEDOT_GPTMS 1% SEM image under 100 times magnification. (**C**) PEDOT_GPTMS 2% SEM image under 250 times magnification. (**D**) PEDOT_GPTMS 2% SEM image under 100 times magnification. (**E**) PEDOT_GPTMS 3% SEM image under 250 times magnification. (**F**) PEDOT_GPTMS 3% SEM image under 100 times magnification. (**G**) Distribution of the different porosities dimensions in all of the piezoresistive sponges investigated.

In view of their subsequent infiltration by an elastomeric polymer, the qualitative porous structure and morphology of the PEDOT:PSS sponges was studied to assess pore morphology and pore interconnection, which is the most important feature for the fabrication of the final flexible transducer. The freeze-dried samples presented porosities were derived from the sublimation of ice crystals, as demonstrated by the SEM images reported in Figure 2.

The SEM images were analysed using the ImageJ software which allows for the quantification of pore areas.

The pores’ area distribution for each percentage and volume/volume of GPTMS has been presented in Figure 2G, arranged as accorded by a previous work [37].

All samples were characterized by porosities mainly within the range of 200–600 µm^2^; as it can be seen, pores of such dimensions represented 50.8% of the total porosities for PEDOT_GPTMS 1%, 46.7% for PEDOT_GPTMS 2%, and 64.5% for PEDOT_GPTMS 3%. Sponges containing 3% *v*/*v* of GPTMS stuck out, exhibiting the largest number of small porosities. This observation is backed up by the literature which affirms that higher concentrations of crosslinker in PEDOT:PSS solutions leads to the generation of freeze-dried sponges that showcase porosities with diminished surface areas [38,39]. The agreement with results and trends already observed in other literature confirms the goodness of the methods employed in the first part of the study.

### 3.2. Stability Evaluation

The stability test aimed to establish which percentage of volume/volume of crosslinker yielded a more stable polymeric sponge in the simulated physiological conditions. This investigation was carried out in view of the potential applications of the native conductive sponges, given their very interesting properties. Possible applications could be in use as scaffolds for tissue engineering and/or for regenerative medicinal purposes that require electrical stimulation during the culturing of cells, such as in muscular, cardiac, or nervous tissue [40,41].

**Figure 3:**
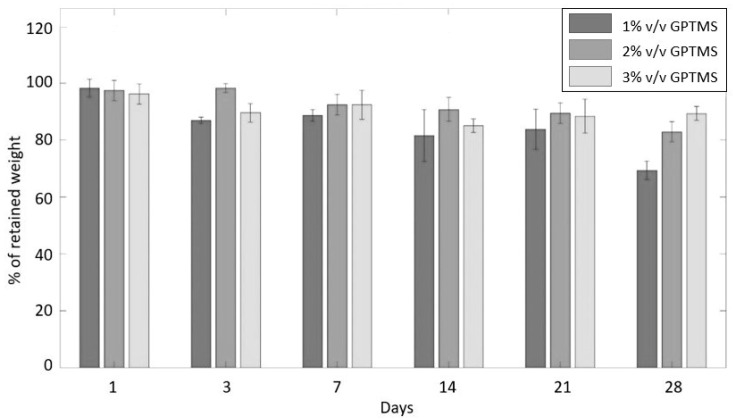
Results of the stability study for PEDOT:PSS sponges crosslinked with three different percentages of volume/volume of GPTMS.

In Figure 3, the percentage of weight retained by the samples at each time interval of the investigation is displayed. The earlier time intervals (1, 3, and 7 days) display a similar behaviour in terms of the weight loss showcased by the three classes, where no great differences can be underlined. The stability of PEDOT_GPTMS 2% and PEDOT_GPTMS 3% is maintained at a nearly constant rate even after 2, 3, and 4 weeks of the test, while PEDOT_GPTMS 1% clearly shows a downward trend. As the test protracts in time, a growing number of polymeric chains leaves the sponges to dissolve in the buffer saline medium due to the intrinsic hydrophilic nature of the polymer. These experimental data are largely supported by the literature, where GPTMS is used as a crosslinker specifically to establish additive links between polymeric chains and therefore preventing their leaking, adding stability to the final structure of the polymer [37,42,43,44,45]. However, these experimental data highlight the potential of GPTMS in increasing the PEDOT:PSS stability even in low concentrations. In fact, previous studies have demonstrated this effect for higher concentrations of GPTMS [38,42].

### 3.3. Electromechanical Characterization under Compression

The most prominent feature that had to be investigated in the PEDOT:PSS sponges was their piezoresistive behaviour during the compressive loading, with respect to the potential sensing applications. Before any compression was applied, an electrical acquisition was performed through the voltage divider to measure the initial resistance of every sample. The collected data are shown in Table 3.

It can be pointed out that an increasing percentage of volume/volume of GPTMS caused the sample to increase its native resistance, yielding an overall less conductive material. The combination of crosslinked PEDOT:PSS sponges with a silicone elastomer also led to a reduction of the final material conductivity. The mechanical performance of the PEDOT:PSS sponges was evaluated through four compressive cycles, each time setting a suitable maximum deformation applied on the sample that would avoid the generation of excessive internal stresses within the material. λ_biax_ is represented on the *x*-axis, which is the axial deformation by compression that the sample endures, calculated as $\frac{h_{0}-h}{h_{0}}$ (*h*_0_ = initial thickness of the specimen under test and *h* = thickness of the compressed specimen), while the stress experienced by the sample is depicted on the *y*-axis. The symbol lambda has been chosen because the axial deformation is only one component of the strain endured by the sample, the other being radial extension in the perpendicular plane.

**Figure 4:**
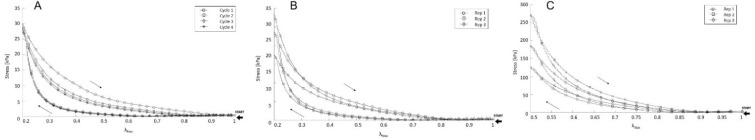
Arrows indicate the direction of advancement of the curve from starting point (**A**) Compressive stress–lambda curve for PEDOT_GPTMS 1% under the cyclic compression with max deformation of 80%, showing a distinctively different behaviour during the first cycle of loading. (**B**) Compressive stress–lambda curve for PEDOT_GPTMS 1% under the cyclic compression and max deformation of 80%, the last three cycles were mediated and the test was carried out in triplicates. (**C**) Compressive stress–lambda curve for PEDOT_GPTMS 1%/PDMS under the cyclic compression and max deformation of 50%, the last three cycles were mediated and the test was carried out in triplicates.

As shown in Figure 4A, the first loading curve distinctly separates itself from the subsequent three compressive curves while the four unloading curves are reasonably comparable; the material significantly changes its response after the first application of compressive stress: this phenomenon could be something similar to the Mullins effect observed in filled rubbers and hydrogels [46,47], where the internal structure of the material is permanently changed during the first application of the load, causing it to react differently but also much more repeatably in the subsequent cycles. The effect could be attributed to microstructural ruptures or changes such as a rearrangement of polymeric chains; even if our native conductive sponges are not very similar to filled rubbers and hydrogels, this could also occur in the PEDOT_GPTMS samples. From the graphs, it is evident that the material displays a hysteretic behaviour. Its response does not depend only on its current state, but also on its past history. We deduce that the material does not possess pure elastic properties, because as the percentage of hysteresis (area comprised between the loading and unloading curves, calculated as a percentage of the loading area) increases, the sample behaves less and less elastically. These last remarks on the compressive mechanical behaviour are valid for all of the specimens tested during the study, with similar curve trends for both the PEDOT:PSS native sponges (PEDOT_GPTMS 1%, PEDOT_GPTMS 2% and PEDOT_GPTMS 3%) and their combination with PDMS (PEDOT_GPTMS 1%/PDMS). However, it is interesting to point out the differences between the tested samples in terms of percentage of hysteresis and σ_max_ (the stress experienced at maximum strain), which were calculated from the stress–lambda curves, excluding the first compressive cycle and mediating the subsequent three cycles (Figure 4B,C).

The calculated mechanical parameters are summarized in Table 4.

A trend can be inferred by the data: the higher the percentage of volume/volume of GPTMS, the higher the σ_max_. The maximum stress can be considered a measure of the material resistance and toughness, where the increment being due to the greater presence of crosslinkers could be justified, considering that the PEDOT_GPTMS 3% sample shows a decrease in the porosities’ dimensions. Compared to the other samples, the PEDOT_GPTMS 3% is a less porous or “bulkier” material, therefore it offered higher resistance to the application of deformation. With respect to hysteresis, it roughly remained constant among the different cases, proving that the elastic properties of the material did not improve with the increase of crosslinker. As for the sample PEDOT_GPTMS 1%/PDMS, the addition of a silicone elastomer certainly improved the overall mechanical behaviour of the polymeric material. Hysteresis was significantly reduced thanks to the newly acquired elastic properties ascribable to the rubber component. Furthermore, the samples manifest a boost in the σ_max_ experienced during the compressive load: this could be attributed to a rise in the material toughness and resistance, most likely due to the fact that all of the porosities initially present in the sponge were filled with silicone, converting the porous sponge into a bulk sample.

**Figure 5:**
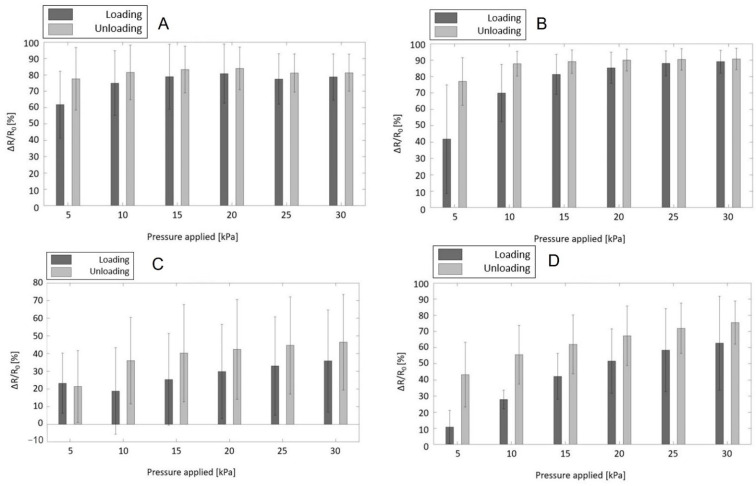
The magnitude of intrinsic resistance variation displayed by the samples under investigation, in response to pressure triggers ranging from 5 to 30 kPa: (**A**) PEDOT_GPTMS 1%; (**B**) PEDOT_GPTMS 2%; (**C**) PEDOT_GPTMS 3%; and (**D**) PEDOT_GPTMS 1%/PDMS.

Moving on to the piezoresistive properties, the percentage of sample resistance variation (compared to their initial resistance value) was evaluated while the samples were submitted to cyclic compression tests at a velocity of deformation of 10 mm/min (dynamic behaviour). These results are depicted in the following bar plots (Figure 5A–C) for each specimen under test during both the loading and unloading phases of the mechanical compression and for increasing values of applied pressure, ranging from 0 to 30 kPa.

These plots clearly show that all samples behaved better when the load was gradually released, yielding a greater and therefore more noticeable variation of resistance: in the same fashion as the majority of piezoresistive sensors [48,49,50], the material displays electrical hysteresis and therefore, a non-linear electrical behaviour. It can be suggested that, once the sample has already been stressed, its sensitivity towards the application of pressure is somewhat enhanced, probably due to permanent internal structure changes during the loading phase. Continuing on and observing the different piezoresponsive behaviours among the three percentages of volume/volume of crosslinker, an increasing concentration of GPTMS in the sponges caused a decrease in terms of response intensity, which could be compared to the measure of sensitivity: $\frac{∆R}{R_{0}}$ fell down from 80–90% for the lower concentrations investigated (PEDOT_GPTMS 1% and PEDOT_GPTMS 2%) to a maximum of 40% for the highest concentration (PEDOT_GPTMS 3%). The PEDOT_GPTMS 3% also displayed higher values of standard deviations compared to the lower concentrations of GPTMS, proving itself to be insufficiently repeatable. The observed poor sensitivity and repeatability led to the PEDOT_GPTMS 3% sample being discarded from further investigation. Another important aspect of the piezoresponsive behaviour of the material can be highlighted in its working range. When stimulated within the limits of its working range with increasing pressure, the material subsequently enhances the intensity of the response. This allows for a quantification of the stress based on the resistance variation measured. PEDOT_GPTMS 1% exhibited a working range of 0–10 kPa while PEDOT_GPTMS 2% reached up to 25 kPa. Once stressed over the upper limit of its working range, the material practically yielded an unvaried response that does not allow for a precise conversion of electrical stimuli into physical. Nonetheless, it could still be employed as a pressure switch that aims to detect the presence or absence of applied pressure without punctual pressure sensing. Despite the narrower working range, the PEDOT_GPTMS 1% sample responded similarly during the loading and unloading phases, showing a more homogeneous/coherent behaviour. Moreover, the material also proved itself to be less fragile, both visually and to manual handling (see Supplementary Information Figure S2). These findings led to applying it with the effort to produce a material that could combine mechanical flexibility and electrical conductivity, pairing a silicone-based elastomer with the piezoresistive sponges. From the data in Table 3, the newly obtained flexible transducer (PEDOT_GPTMS 1%/PDMS) showed a higher intrinsic resistance value than the native conductive polymer, and therefore a diminished electrical conductivity. This could be expected, given that the PEDOT:PSS network, which allows for the conduction of electrons, becomes intricately intertwined with an insulating polysiloxane. Regarding the electromechanical characterization, the native PEDOT:PSS sponges partially lose their initial sensitivity when combined with PDMS, reaching a maximum $\frac{∆R}{R_{0}}$ of 70% (Figure 5D).

In the range of investigated pressures (0–30 kPa), the sample PEDOT_GPTMS 1%/PDMS showed a proportionally increasing response; therefore, its working range extended further than 30 kPa. As previously mentioned, the material behaved better during the unloading phase. Compared with the native PEDOT_GPTMS 1%, the flexible transducer exhibited a lower native conductivity, broader working range (allowed by its better elasticity and overall mechanical properties), and similar intensity of piezoresistive response, hence sensitivity, towards external compressive stress. Therefore, pressure sensing and pressure switch applications remain valid, as long as the stresses involved in the sensing applications match the specific limits of the working range.

### 3.4. Determination of Surface Wettability

Given the combination of two very different materials, with respect to superficial wettability, the flexible transducer was submitted to water contact angle measurements in order to uncover the final characteristics. The control was native PDMS material not combined with PEDOT:PSS; native conductive sponges (PEDOT_GPTMS 1%, PEDOT_GPTMS 2% and PEDOT_GPTMS 3%) were left out of this investigation due to their strongly hydrophilic nature. Both tested samples exhibited a hydrophobic behaviour, with the PDMS contact angle value around 116.3° ± 1.2° and the flexible transducer angle around 121.6° ± 9.3° (see Supplementary Information Figure S3). No significant differences were found in terms of surface wettability when a network of hydrophobic silicone elastomer was intertwined with a strongly hydrophilic one, which could be attributed to the fabrication technique. The PEDOT:PSS sponge was immersed in a solution of PDMS precursors in order to be infiltrated by the elastomeric material; the surface remained in close contact with silicone for approximately 5 min and SEM images (see Supplementary Information Figure S5) display a considerable presence of PDMS on the sample surface, despite the formation of electrically-insulating films being largely prevented (see Supplementary Information Figure S4). Therefore, we observe that the hydrophobic silicone portion of the final material dominates over the hydrophilic one.

### 3.5. Cytotoxicity Evaluation

The flexible transducer PEDOT_GPTMS 1%/PDMS underwent an eluate test following the protocol described in the ISO 10993:5-2009 [51] document (Biological evaluation of medical devices—Part 5: Tests for in vitro cytotoxicity) with NIH 3T3 cells to evaluate any potential cytotoxicity that could discourage interactions with biological tissues. The cell line NIH 3T3, an established cell line for preliminary tests evaluating the cytocompatibility of devices and materials, as indicated in ISO 10993-5:2009 [51], was isolated from a mouse NIH Swiss embryo.. Incubation with the eluate test from PEDOT_GPTMS 1%/PDMS samples showed higher fluorescence values compared with the native PDMS and control (regular cellular culture medium): 4396 ± 173.83 for native silicone and 4888.92 ± 62.35 for the flexible transducer, as opposed to the control regular medium 4237.33 ± 224.60. Higher values of fluorescence indicate a higher presence of vital, healthy cells. These preliminary biological characterizations indicate that the material does not exert any cytotoxic activity against cells; therefore, it would not be unlikely to consider its employment for in vivo. PEDOT:PSS has already been investigated for its supposed cytotoxicity due to residual EDOT monomers and the possible negative interaction between PSS molecules and cells [52,53], but the results ultimately rejected the hypothesis. Furthermore, PDMS is an established biocompatible polymer [54,55], widely used in several biomedical applications.

### 3.6. Estimation of Potential Anti-Biofilm Properties

**Figure 6:**
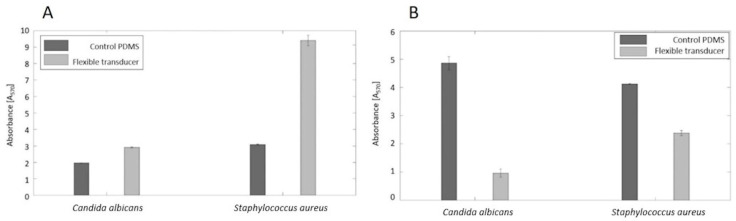
(**A**) The metabolic activity estimation for planktonic cells through absorbance measurements after the MTT reduction assay for PEDOT_GPTMS 1%/PDMS compared with native PDMS. (**B**) The biofilm biomass estimation through absorbance measurements after CV staining for PEDOT_GPTMS 1%/PDMS compared with the native PDMS.

**Figure 7:**
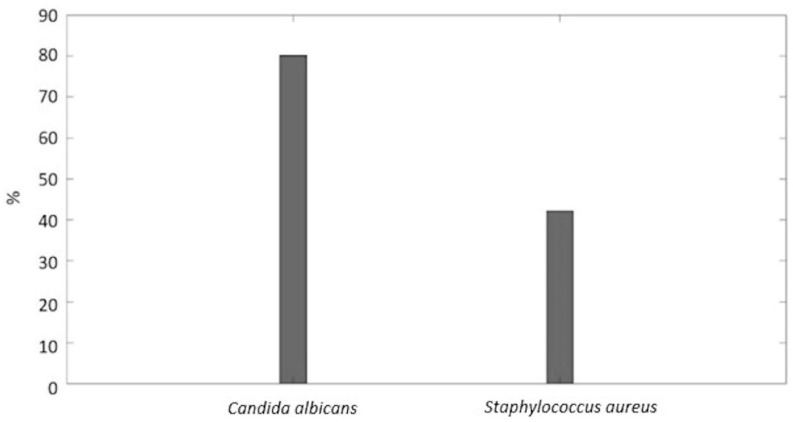
The percentages of bacterial inhibition calculated through the CV absorbance measurements for the PEDOT_GPTMS 1%/PDMS material.

The PEDOT_GPTMS 1%/PDMS biomaterial has undergone two different anti-biofilm assays in order to evaluate if its surface supported, promoted, or in any way discouraged bacterial adhesion, in view of the potential in vivo sensing applications, which are among the wide range of applications for soft robotics. We determined the tests to be useful, as PDMS is a biomaterial widely used for in vivo applications but unfortunately prone to failure given its natural hydrophobicity, leading to bacteria adhesion and the consequent colonization of the surface by biofilm formations [56,57]. Each test was carried out using both a bacterial species (*Staphylococcus aureus*) and a fungus (*Candida albicans*). Experiments were carried out in triplicates and reported as the mean ± standard deviation. In order to assess the ability of the developed materials to increase/decrease the bacterial adhesion and consequent biofilm, PEDOT_GPTMS 1%/PDMS material was tested by using two anti-biofilm assays. The biofilm biomass and metabolic activity of planktonic cells are characteristics that can both indicate an increase/decrease of the bacterial adhesion and consequent biofilm production on the material surface. Figure 6A,B report the absorbance values (A_570_) at 570 nm, collected from the MTT reduction and crystal violet (CV) assays, respectively. The assays were also performed on the native PDMS as a control. The CV results showed a lower absorbance value than the control for both *Staphylococcus aureus* (0.96 ± 0.23) and *Candida albicans* (2.38 ± 0.09), meaning that the tested material retained less biofilm on its surface. The MTT reduction assay also yielded higher absorbance values for the flexible transducer compared with the control for *Staphylococcus aureus* (2.92 ± 0.03) and *Candida albicans* (9.40 ± 0.32), indicating higher presence of vital planktonic cells in the supernatant and therefore the tendency of bacterial cells to remain in an isolated state instead of adhering to the surface to form biofilm. Using the absorbance values retrieved from the CV assay, the percentages of bacterial inhibition of the material, compared with the control, were calculated following Equation (5), and are shown in Figure 7. There was an 80.2% inhibition against *Candida albicans* and 42.2% against *Staphylococcus aureus*. These findings seem promising, giving the prospect that the final material could exert anti-biofilm and anti-adhesive properties to an extent, therefore not promoting bacterial adhesion and colonization on its surface when tested against two of the most common strains of fungus (*C. albicans*) and bacterium (*S. aureus*) responsible for nosocomial infections. More in-depth biological characterizations should be performed to test the material for possible in vivo sensing applications. The surface properties of the material and its interaction with bacteria (or cells in general) could be tailored through the means of surface functionalization, employing specific anti-fouling and/or anti-microbic biomolecules that could improve or introduce useful features for the applications under consideration.

## 4. Conclusions

In this study, we have thoroughly characterized and investigated native PEDOT:PSS sponges crosslinked with different percentages of volume/volume of GPTMS. The sponges were obtained through freeze-drying, which is a simple and versatile technique allowing for different shapes and sizes, also avoiding the use of organic solvents. When tested for stability over time intervals of up to four weeks, the samples characterized by larger presences (PEDOT_GPTMS 2% and PEDOT_GPTMS 3%) of GPTMS did not show a relevant amount of weight loss, as was expected, while the PEDOT_GPTMS 1% samples clearly revealed a downward trend in the percentage of retained weight. A greater presence of GPTMS also increased the number of smaller porosities found in the sponges according to the results of the morphological analysis performed. Regarding the piezoresistivity, the native PEDOT:PSS sponges’ response to external pressure triggers proved itself to be linear within the limits of their specific working range. Moreover, the lower concentrations of GPTMS tested (PEDOT_GPTMS 1% and PEDOT_GPTMS 2%) displayed a greater sensitivity and repeatability, but a narrower working range than in PEDOT_GPTMS 3%; the greatest concentration of GPTMS also showed an unacceptable level of fragility and response variability. We deduced that the amount of GPTMS added is a variable factor that helps to modulate the final material stability, morphology and piezoresistive properties, and the amount could be adjusted to the specific sensing application under consideration; the final step assessed would involve the acquisition of a precise calibration curve of the sensor. Given the more homogeneous response showed during the loading and unloading trials, the enhanced sensitivity towards pressure triggers, and the less fragile behavior displayed by PEDOT_GPTMS 1%, it was decided to interconnect this particular piezoresistive sponge with an elastomeric silicone network, yielding PEDOT_GPTMS 1%/PDMS. The flexible transducer is characterized by an improved mechanical elasticity and resistance, displaying a much lower mechanical hysteresis and a greater value of maximum stress endured during the mechanical tests. The piezoresistivity is only slightly affected even though the native conductivity is lowered, and the response variability increases. The magnitude and linearity of the material response to compression triggers is not drastically reduced. Moreover, the interconnection with an elastomeric network yields a broader working range for the final transducer. Therefore, we determine that pressure sensing and pressure switch applications for the piezoresistive sponges are still valid for the flexible transducer developed in this study, surely after gaining characteristics for the sensor. PEDOT_GPTMS 1%/PDMS has also been submitted to preliminary biological characterizations, a cytotoxicity assay, and two anti-biofilm assays. The results indicate no damage of cells and a mild anti-biofilm effect exerted on the biomaterial surface, thus showing promising potential for further in vivo sensing applications. Its surface properties and interaction with bacteria (or cells in general) could be tailored by means of surface functionalization, employing specific molecules such as anti-fouling or anti-microbic moieties that could improve or introduce useful features for the applications that are under consideration.

## Figures and Tables

**Table 1:** The set of parameters for the voltage divider circuit used in every electromechanical test and performed on all samples under investigation.

% *v*/*v* GPTMS	Repetition	V_in_ [V]	R_c_ [KΩ]
1%	1–2–3	5	56
2%	1–2–3	5	220.5
3%	1–2	5	1200
	3		395
1% + PDMS	1	5	6860
	2–3		1200

V_in_ = input voltage; R_c_ = known resistance value for the resistor in series with our sample.

**Table 2:** The set of parameters used for every cyclic compression test performed on all samples under investigation.

% *v*/*v* GPTMS	n° of Cycles	ε_max_ [%]	V_def_ [mm/min]
1%	4	50	0.5–10
		80	
2%	4	80	0.5–10
3%	4	80	0.5–10
1% + PDMS	4	50	0.5–10

ε_max_ = maximum deformation applied to the sample during mechanical compression; V_def_ = velocity of deformation.

**Table 3:** The initial intrinsic resistance value calculated for each sample under investigation through voltage divider circuit acquisitions.

% *v*/*v* GPTMS	V_in_ [V]	Rep	R_c_ [kΩ]	R_sample_ [kΩ]
1%	5	1–2–3	56	100.53 ± 62.46
2%	5	1–2–3	220.5	464.74 ± 107.37
3%	5	1–2	1200	1942.5 ± 1467.1
		3	395	
1% + PDMS	5	1	6860	52,138 ± 69,175
		2–3	1200	

V_in_ = input voltage; rep = number of repetitions of the experiment; R_c_ = known resistance value for the resistor in series with our sample; R_sample_ = resistance value calculated as mean ± standard deviation of the three measurements performed on the different samples for each type of biomaterial under investigation.

**Table 4:** The maximum stress endured and hysteresis calculated for each sample under investigation through mechanical cyclic compressive tests.

% *v*/*v* GPTMS	ε_max_	V_def_ [mm/min]	σ_max_ [kPa]	% Hysteresis
1%	80%	0.5	15.49 ± 3.68	74.36 ± 3.42
2%	80%	0.5	22.17 ± 15.09	80.40 ± 9.51
3%	80%	0.5	49.51 ± 20.07	64.18 ± 33.05
1% + PDMS	50%	0.5	129.08 ± 48.32	24.32 ± 5.37

ε_max_ = maximum deformation applied to the sample during mechanical compression; V_def_ = velocity of deformation; σ_max_ = maximum stress endured by the sample during mechanical compression.

## Data Availability

Not applicable.

## Supplementary Materials

The following supporting information can be downloaded at: https://www.mdpi.com/article/10.3390/jfb13030135/s1, Figure S1: Experimental setup employed to perform the electromechanical characterizations on all samples tested; Figure S2: Samples after four cycles of the compressive mechanical test, showing an increasing loss of integrity and original shape for higher concentrations of GPTMS: (A) PEDOT_GPTMS 1%, (B) PEDOT_GPTMS 1%/PDMS, (C) PEDOT_GPTMS 2%, (D) PEDOT_GPTMS 3%; Figure S3: WCA value and drop images for the native PDMS (left) and PEDOT_GPTMS 1%/PDMS (right); Figure S4: Frontal photo of (A) PEDOT_GPTMS 1%, (B) PEDOT_GPTMS 1%/PDMS, showing that no insulating PDMS film forms on the surface; Figure S5: SEM images of PEDOT_GPTMS 1%/PDMS.
