# Supplementary material for: Development of an Innovative Soft Piezoresistive Biomaterial Based on the Interconnection of Elastomeric PDMS Networks and Electrically-Conductive PEDOT:PSS Sponges
Source: J Funct Biomater. 2022 Aug 29;13(3):135. doi: 10.3390/jfb13030135 (PMC9500767; doi:10.3390/jfb13030135)
Supplement: Supplementary file 1 [file jfb-13-00135-s001.zip › jfb-1837783-supplementary.pdf]

Development of an innovative soft piezoresistive biomaterial based on the interconnection of elastomeric PDMS networks and electrically-conductive PEDOT:PSS sponges

Maria Antonia Cassa [1,2], Martina Maselli [3,4], Alice Zoso [1,2], Valeria Chiono [1,2,5], Letizia Fracchia [6], Chiara Ceresa [6], Gianluca Ciardelli [1,2,5], Matteo Cianchetti [3,4] and Irene Carmagnola [1,2,*]

1 Politecnico di Torino, Department of Mechanical and Aerospace Engineering, Corso Duca degli Abruzzi 24, 10129 Torino, Italy
2 Politecnico di Torino, Polito BIOMed Lab, Corso Castelfidardo 30/a, 10129 Torino, Italy
3 Scuola Superiore Sant'Anna, The BioRobotics Institute, Viale Rinaldo Piaggio 34, Pontedera, 56025 Pisa, Italy
4 Scuola Superiore Sant'Anna, Department of Excellence in Robotics & AI, Piazza Martiri della Libertà 33, 56127 Pisa, Italy
5 National Research Council, Institute for Chemical and Physical Processes (CNR-IPCF), 56124 Pisa, Italy
6 Department of Pharmaceutical Sciences, Università del Piemonte Orientale "A. Avogadro", Largo Donegani 2, 28100 Novara, Italy
* Correspondence: irene.carmagnola@polito.it

N° of PAGES 3

N° of FIGURES 5

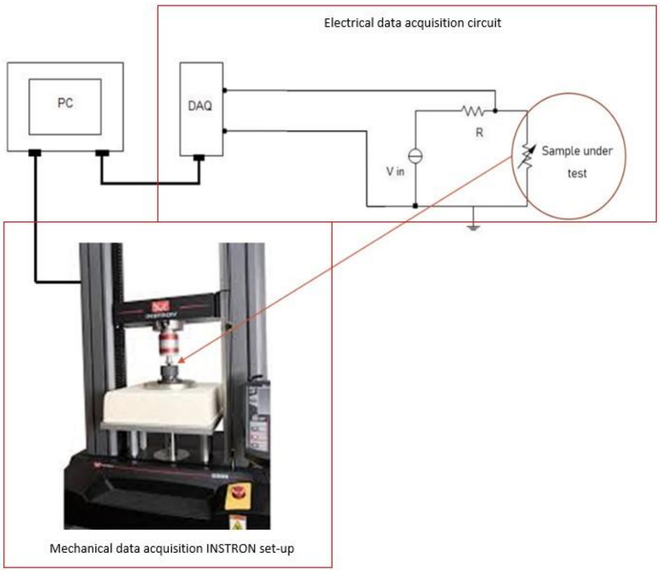

Figure S1. Experimental set-up employed to perform the electromechanical characterizations on all samples tested.

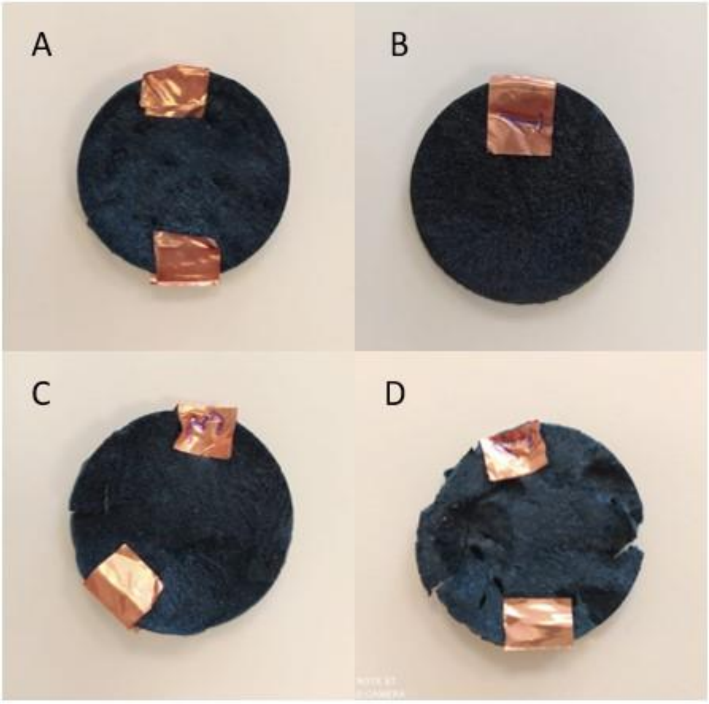

Figure S2. Samples after 4 cycles of compressive mechanical test, showing an increasing loss of integrity and original shape for higher concentrations of GPTMS (A) PEDOT_GPTMS 1% (B) PEDOT_GPTMS 1% / PDMS (C) PEDOT_GPTMS 2% (D) PEDOT_GPTMS 3%.

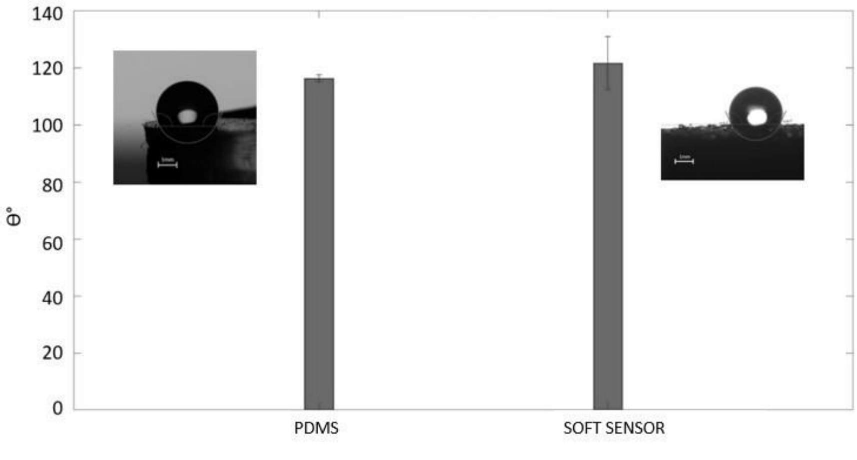

Figure S3. WCA value and drop images for native PDMS (left) and PEDOT_GPTMS 1% / PDMS (right).

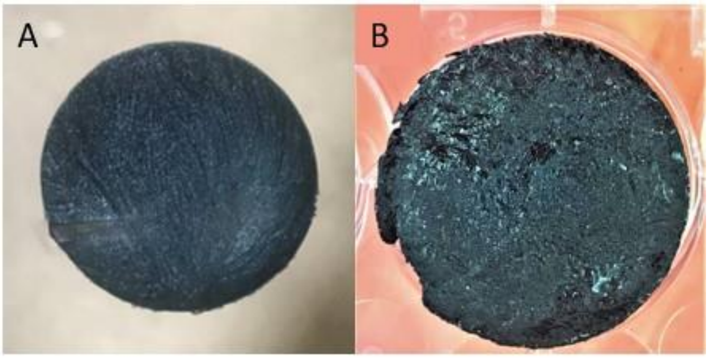

Figure S4. Frontal photo of (A) PEDOT_GPTMS 1% (B) PEDOT_GPTMS 1% / PDMS showing that no insulating PDMS film forms on the surface.

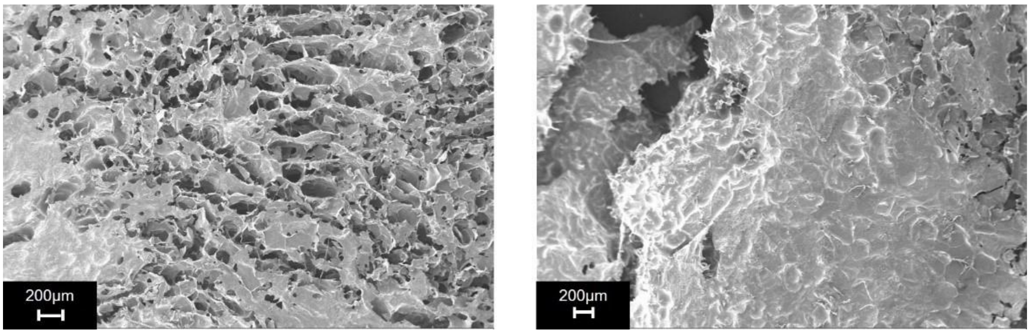

Figure S5. SEM images of PEDOT_GPTMS 1% / PDMS obtained through (left) 5 min immersions in two separate wells (right) 48h leave-in method.
